# COGNATE: **co**mparative **g**e**n**e **a**nno**t**ation charact**e**rizer

**DOI:** 10.1186/s12864-017-3870-8

**Published:** 2017-07-17

**Authors:** Jeanne Wilbrandt, Bernhard Misof, Oliver Niehuis

**Affiliations:** 1 Zoologisches Forschungsmuseum Alexander Koenig (ZFMK), Zentrum für Molekulare Biodiversitätsforschung (zmb), Bonn, Germany; 2 Abteilung Evolutionsbiologie und Ökologie, Albert-Ludwigs-Universität Freiburg, Institut für Biologie I (Zoologie), Freiburg, Germany

**Keywords:** Comparative genomics, Protein-coding genes, Gene annotation, Gene repertoires, Gene structure, Standardization

## Abstract

**Background:**

The comparison of gene and genome structures across species has the potential to reveal major trends of genome evolution. However, such a comparative approach is currently hampered by a lack of standardization (e.g., Elliott TA, Gregory TR, Philos Trans Royal Soc B: Biol Sci 370:20140331, 2015). For example, testing the hypothesis that the total amount of coding sequences is a reliable measure of potential proteome diversity (Wang M, Kurland CG, Caetano-Anollés G, PNAS 108:11954, 2011) requires the application of standardized definitions of coding sequence and genes to create both comparable and comprehensive data sets and corresponding summary statistics. However, such standard definitions either do not exist or are not consistently applied. These circumstances call for a standard at the descriptive level using a minimum of parameters as well as an undeviating use of standardized terms, and for software that infers the required data under these strict definitions. The acquisition of a comprehensive, descriptive, and standardized set of parameters and summary statistics for genome publications and further analyses can thus greatly benefit from the availability of an easy to use standard tool.

**Results:**

We developed a new open-source command-line tool, COGNATE (**Co**mparative **G**e**n**e **A**nno**t**ation Charact**e**rizer), which uses a given genome assembly and its annotation of protein-coding genes for a detailed description of the respective gene and genome structure parameters. Additionally, we revised the standard definitions of gene and genome structures and provide the definitions used by COGNATE as a working draft suggestion for further reference. Complete parameter lists and summary statistics are inferred using this set of definitions to allow down-stream analyses and to provide an overview of the genome and gene repertoire characteristics. COGNATE is written in Perl and freely available at the ZFMK homepage (https://www.zfmk.de/en/COGNATE) and on github (https://github.com/ZFMK/COGNATE).

**Conclusion:**

The tool COGNATE allows comparing genome assemblies and structural elements on multiples levels (e.g., scaffold or contig sequence, gene). It clearly enhances comparability between analyses. Thus, COGNATE can provide the important standardization of both genome and gene structure parameter disclosure as well as data acquisition for future comparative analyses. With the establishment of comprehensive descriptive standards and the extensive availability of genomes, an encompassing database will become possible.

**Electronic supplementary material:**

The online version of this article (doi:10.1186/s12864-017-3870-8) contains supplementary material, which is available to authorized users.

## Background

As more and more sequenced genomes become available, studying the commonalities and differences in the structure of genes and genomes has become an exciting and a rapidly expanding research field. Examples of comparative studies of intron size are those published by Yandell et al. [1], Moss et al. [2], and Zimmer et al. [3], who found that intron length evolution behaves clock-like, that ancient bursts of repetitive elements can be responsible for an unusual intron length distribution, and that there is a trend towards shorter introns in the evolution of land plants, respectively. These studies were restricted to a rather unrepresentative selection of animal, fish, and plants species, respectively, due to the lack of genome sequences. Studies with much larger species numbers and a broader taxonomic coverage are becoming feasible.

Elliott & Gregory [4] recently published a seminal meta-analysis of the genome and gene summary statistics of animals, land plants, fungi, and ‘protists’, relying on 521 species. The large number of species and genomes considered in their analysis allowed the authors to robustly detect statistical trends in genome evolution, such as a positive correlation between genome size and both gene and intron content, while taking phylogenetic relationships into account. These trends have been previously observed (e.g., [5, 6]), but were based on a much smaller taxonomic sampling. Yet, despite the evidently improved availability of sequenced genomes, Elliott & Gregory [4] struggled with a lack of standards in the disclosure of genome characteristics when compiling data for their analyses; they evaluated 28 parameters of the genomes of 521 species (see Supplement of [4]), for which only 48% of all possible values were provided in the publications to the respective genomes (cf. Fig. 2) and thus available for the meta-analysis.

The lack of standardization in the publication of gene structure characteristics is a general problem. Not only are some basic gene content and structure statistics frequently presented in a non-standardized manner, it often remains unclear whether or not terms describing gene structure were consistently applied to achieve comparability between analyses. For example, gene counts may or may not be inferred from tallying all predicted transcripts, thus bearing the risk of including alternative transcripts or isoforms as pseudo-replicates in meta-analyses. Furthermore, GC content may be reckoned without respect to IUPAC base-calling ambiguity in the total sequence lengths, which predicates the resulting value on sequencing and assembly quality. Finally, it can be difficult to trace inconsistencies in the use of terms, like ‘exon’ versus ‘coding sequence (CDS)’ despite existing standard vocabularies like the Sequence Ontology [7]. Clearly, comparability and traceability of published data can greatly benefit from standardized analyses of genome organization and gene structure (see also [8]).

A partial explanation for the lack of a standardized analysis and presentation of fundamental genomic features referring to protein-coding genes is a lack of software that infers the desired statistics. Available tool suites like BEDtools [9], genomeTools [10], AEGeAN,^1^ and gfftools^2^ are mostly intended for processing rather than describing annotations. While various programming libraries, such as BioPerl^3^ and SeqAn [11] provide suitable methods, their usage is demanding to researchers without programming experience and fosters the development of custom scripts by researchers with programming skills. The former likely limits the number of scientists who can infer the desired statistics, while the latter increases the risk of inferring incompatible results due to errors and/or misconceptions in analyses and definitions. Thus, there is a need for easy to use software that provides the facility to examine genome annotations for a wealth of structural features of the protein-coding gene repertoire in a concise way and that provides basic and standardized statistics as well as results suitable for downstream applications.

Here we present the tool COGNATE, a ***Co*** *mparative* ***G*** *e* ***n*** *e* ***A*** *nno* ***t*** *ation Charact* ***e*** *rizer.* It fills the above identified gap of software for structural characterization of the annotated protein-coding gene repertoire of a genome. COGNATE allows a quick and easy extraction of basic genome features and gene repertoire data; it is thus a tool to primarily describe a genome and its annotated protein-coding gene repertoire, which is an essential prerequisite for comparative analyses. Given the ongoing genome sequencing efforts, especially by large consortia like 10 k [12] and i5k [13], we see an increasing demand for a standardization of large-scale comparisons of genome and gene structure.

## Implementation

**Fig. 1:**
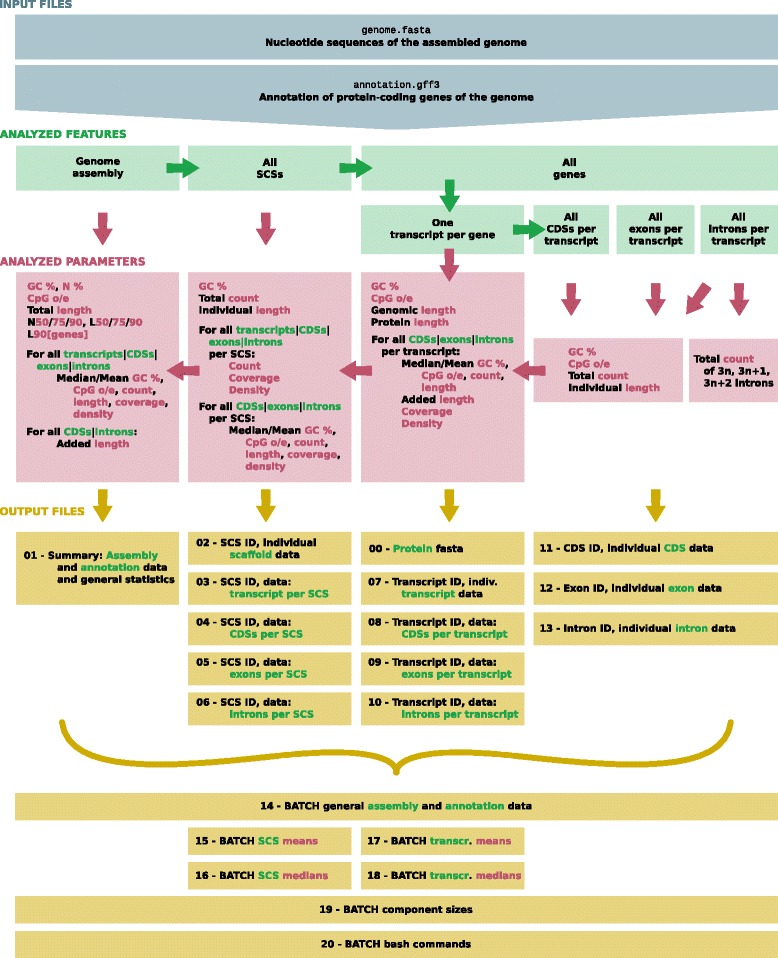
Overview of the information flow in the software package COGNATE. The Perl script COGNATE requires two files per run as input (blue): a fasta file containing the assembled nucleotide sequences and a GFF3 file with the protein-coding gene annotation information. The input (blue) is used to analyze genomic and genic features (green) on the level of assembly, SCSs, transcripts, CDSs, exons, and introns. Each complex of analyzed features is evaluated individually and the analyzed parameters are condensed in a step-wise manner by calculating means and medians (red). As output (yellow), 21 files are generated, of which all except two are in TSV format (the exceptions are: 00, protein fasta; 20, bash commands). The output files are split according to the analyzed features and parameters. All data files (02–13) are ordered by the ID of the respective feature. BATCH files (14–20) contain one entry line per genome and thus data of multiple COGNATE runs to facilitate direct comparisons of genomes. CDS: CoDing Sequence; GFF: Generic Feature Format; SCS: Scaffold or Contig Sequence; TSV: Tab-Separated Values

With COGNATE, we promote a tool to simultaneously analyze a given protein-coding gene annotation and the corresponding assembled sequences of a genome, here referred to as scaffold or contig sequence (SCS). An overview of the software’s input, work flow, analyzed parameters, and output is visualized in Fig. 1. A complete list of analyzed parameters is given in Additional file 1, a glossary with the definitions of all terms used in this publication and by COGNATE is provided in Additional file 2.

COGNATE requires as input: (1) a gff file in GFF3 format^4^ containing the annotation of protein-coding genes; (2) a fasta file, containing the corresponding genomic nucleotide sequences, which are exploited to infer the length, GC content, and amino acid sequences of the assembled SCSs and of the predicted protein-coding genes, respectively. The gene annotation has to include at least the features ‘gene’, ‘mRNA’, and ‘exon’, as provided by, for example, BRAKER1 [14] and MAKER2 [15]. Thus, the analysis of partial and pseudogenes depends on their annotation in the analyzed gff file; non-coding genes (i.e., genes without mRNA) are not considered in the analysis. Further technical requirements are several standard Perl libraries as well as the GAL::Annotation and GAL::List libraries to allow gff-handling. The latter two libraries are available from the Sequence Ontology Project^5^ and are also included in the COGNATE software package. COGNATE is written in Perl and has been tested under Ubuntu 12.04 and 14.04. COGNATE analyzes one genome at a time. Providing multiple genomes (i.e., a batch) for serial processing is possible with a special input file (see README, Additional file 4). Serial, single-threaded processing leads to a linear relationship of processed genomes and required time. As a gauge, the analysis of the latest *Apis mellifera* gene set (see Results and Discussion), which has a genome size of 250.3 Mb and 10,733 annotated protein-coding genes, takes with COGNATE up to 4 h, using up to 600 MiB RAM. For comparison, COGNATE requires a very similar amount of time for the analysis of the gene set^6^ of *Ixodes scapularis* (genome size: 1765.4 Mb, 20,467 annotated protein-coding genes). A benchmark comparison of COGNATE to other software, such as genomeTools [12], AEGeAN^1^, or gfftools^2^, is not meaningful due to major differences between these software packages in focus and aim. At the moment, no tool yields the wide array of metrics that COGNATE delivers by default.

COGNATE infers the following major metrics (for a full list of the 296 parameters, see Additional file 1):

- summary counts of the analyzed features, including L90pcG^7^, i.e., the number of SCSs needed to cover 90% of all annotated protein-coding genes;
- strandedness of transcripts and features (CDSs, exons, and introns);
- lengths and length statistics (nucleotide/amino acid sequences), including N50/L50, 75/L75, N90/L90;
- intron length distribution [16];
- percental GC content statistics in two different ways, namely
  - using a calculation that explicitly considers IUPAC ambiguity codes (G, C, S per total length excluding N, R, Y, K, M, B, D, H, V);
  - using the previously prevailing calculation of GC per total length, which is inappropriate for genome comparisons due to its dependence on assembly quality;
- statistics of CpG dinucleotide depletion (CpG observed/expected), normalized by C and G content of the respective region [17];
- density statistics (ratio of the length of a feature covered by another, number-wise);
- coverage statistics (ratio of the length of a feature covered by another, length-wise).

In summary, the output parameters can be classified as computations of the eight above major metrics or feature types, some with child types (e.g., added length), of six structural entities (e.g., assembly/annotation, SCSs, introns). In other words, parameters are inferred on several levels. For example, the total count of CDSs in analyzed transcripts is given for the entire assembly as well as on a per transcript basis. For the latter, COGNATE also calculates the mean and median count of CDSs per transcript as well as the mean/median of these medians over all transcripts. As another example, the intron density of a gene is calculated as the total number of introns divided by the length of the gene (i.e., genomic length of the transcript, including introns and exons) and also given as mean/median intron density per gene over the whole annotation. For each gene, only one representative (optionally the longest [default], shortest, or median-length) transcript is evaluated. The analysis is independent of homology hypotheses (i.e., not limited to gene families), thus comprising information on a genome’s entire annotated protein-coding gene repertoire.

As output, COGNATE provides various result tables in TSV format:

- a concise overview (summary) of measured variables;
- lists of all measured variables referring to features of a given SCS, transcript, or individual CDSs, exons, or introns, respectively;
- ‘batch’ files, which contain one line of summary statistics per analyzed genome. There are individual files for general genome data and means and medians of SCS and transcript data, respectively;
- a component size overview (i.e., the added length [in bp] of all coding and intron sequences, respectively), which offers a basis for a comparison of these values with statistics of other genomic features inferred with other tools, for example non-coding elements;

All above specified files (except the one providing an overview) facilitate tests for correlations between parameters within and among genomes. The output files are formatted specifically to allow easy import in statistical software, such as R [18] and SPSS [19]. COGNATE also provides a fasta file (‘analyzed_transcripts’) containing the predicted amino acid sequences inferred from the CDSs of the one analyzed transcript per gene. This file can be used, for example, as input for BUSCO [20] to test for the completeness of the gene set, which is facilitated by the ready-made bash commands supplied in the ‘bash commands’ text file. The generation of all output files can be controlled directly by the user.

The output of COGNATE can be used in manifold analyses, ranging from a descriptive characterization to an in-depth comparative analysis of gene organization across multiple genomes. This is further exemplified in the discussion.

## Results and discussion

It is an essential feature of COGNATE to provide not only descriptive statistics but also the complete primary data, since “an over-reliance on simple summary statistics […] can obscure real biological trends and differences” ([2], p. 1191). Apart from other already mentioned potential applications, COGNATE output can be used to study the variability of gene structure within a genome and to compare it with that in other genomes. In such an instance, the list of transcript features can be exploited to analyze the range of exon lengths, intron lengths, and their distribution over genes of a certain GC content. Another example would be a comparison of GC content in coding and non-coding regions of genes across a genome. Having the characteristics of a gene repertoire at hand, they can be compared to those of other species and used in phylogenomic analyses (e.g., [21]). COGNATE results can also serve as a starting point to find genes of interest and relate them to functions, e.g., looking for very long or short genes or investigating genes containing exactly two CDSs. Hypotheses like ‘Flying birds have shorter introns than birds of non-volant sister lineages due to energetic demands of powered flight’ [6], ‘Evolutionary changes in intron lengths correlate with co-expression of genes’ [22], or ‘Strategies of splice-site recognition are influenced by differences in GC content between exons and introns’ [23] could thereby be tested in more detail. Thus, COGNATE provides data to facilitate downstream analyses, and in addition, provides summary statistics that can help standardizing genome parameter disclosure.

Missing standardization in comparative genomics can easily lead to problems in meta-analyses and consequently result in biased conclusions. As Elliott & Gregory [4] noted during their tremendous effort of data compilation, there are problems of standardization in terms of parameter listing and source disclosure as well as of definitions of descriptive terms. Some of these subtle and sometimes deemphasized problems are elucidated here in more detail to raise and sustain the awareness for them.

**Fig. 2:**
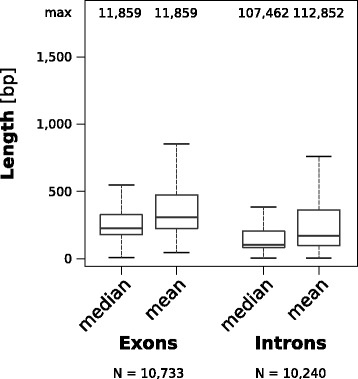
Comparison of means and medians of exon and intron length in the genome of *Apis mellifera*. We applied COGNATE with default options (thus using the longest of each gene’s alternative transcripts) to the genome and gene annotation of *Apis mellifera*, version 4.5. The respective data were downloaded from NCBI^7, 8^ and constitute a RefSeq annotation, predicted by the NCBI Eukaryotic Genome Annotation Pipeline. Shown is the comparison between the mean and median values of the exon length and of the intron length per transcript in bp, respectively. COGNATE considered 10,733 transcripts, comprising of 76,276 exons and 65,543 introns. *N* = 10,733 for mean and median exon lengths and *N* = 10,240 for mean and median intron lengths. The means of exon lengths are 355.32 (medians) and 452.99 (means) bp, means of intron lengths are 613.08 (medians) and 1502.31 (means) bp. The primary data are provided in Additional file 3

One problem in compiling data for meta-analyses are missing values. The data matrix compiled by Elliott & Gregory (Supplement of [1]^12^) contains overall 52% missing values due to incomplete data disclosure by publications or missing entries in databases. This lack of data introduces a potential bias in correlative analyses of genome structures, which has not been systematically investigated. Thus, without in-depth parameter disclosure, the enormous effort of collecting data from open sources for genome and gene structure comparison potentially yields unreliable results. The general distribution of missing data in the matrix compiled by Elliott & Gregory [1] is noteworthy in that the GC content is almost always given while values related to gene structure including intron size values are missing for half of the genomes in the data matrix (see Fig. 2). It is surprising to find that for 38% of the genomes in their dataset no assembly genome size was included in the original publications or databases. To further illustrate the problem of missing data in comparative genomics, we analyzed the genome (version 4.5, downloaded 31 August 2015, from NCBI^8^) and latest protein-coding gene annotation (release 103, downloaded 20 March 2017 from NCBI^9^) of *Apis mellifera*. Compared to the 144 values recorded by COGNATE that can readily be given as a single number, the publications covering the official gene sets 1 [24] and 3.2 [25] offer only eight and nine comparable values, respectively; NCBI offers a report site^10^ for the most recent annotation release (103), where we found 14 comparable values (Additional file 1, sheet 2). The obtained values differ on a small scale (for example, the count of protein-coding genes differs by 5 for a total of circa 10,730), most likely due to the different annotation versions or deviating definitions. Generally, COGNATE can help to mitigate the problem of missing values by easing their acquisition and has the benefit of providing tractable values with a transparent method.

Problems of fuzzy terminology become apparent when, for example, the coding amount (i.e., the total length of protein-coding sequences within a genome) is given in exonic megabases (Mb) (Fig. 2; [4]). Given the functional and structural similarity of exons and CDSs and their often complete overlap in automated annotations, it is an understandable, yet potentially misleading lack of differentiation. In contrast to CDSs, annotated exons can include untranslated regions (UTRs) and stop codons; not every exon is a coding sequence [26]. Most of the automated annotations do not include UTRs, which are difficult to delineate de novo (e.g., [27, 28]); nevertheless, a future project is to include the analysis of UTR annotations in COGNATE. Thus, in this instance, it remains unclear in which form exons and CDSs were evaluated and contributed to a summary statistic. With the above example, we are illustrating why we stress the importance of clear definitions and applications of these to genome and gene structure characterizations. Accordingly, COGNATE differentiates between CDSs and exons, but it can only be as accurate as the given annotation. For a complete list of our definitions, compared to Sequence Ontology terms 11, see the glossary in Additional file 2. The problems of defining a universally needed term such as ‘gene’ (described in [29]) as well as the various ways and needs of gene annotation [30] render the ongoing efforts of finding precise and useful definitions both essential and exacting.

**Fig. 3:**
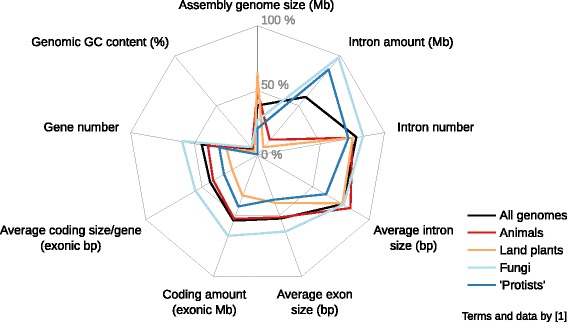
Amount of missing data [%] in nine selected parameters analyzed by Elliott and Gregory [4]. We selected nine parameters evaluated by Elliott and Gregory [4], namely those that are directly comparable to the parameters evaluated by COGNATE. These parameters are: (1) the size of the assembled genome (in Mb); (2) the GC content of the assembled genome in % (COGNATE provides here two values, taking Ns in the sequence into account and excluding them, respectively); (3) gene number (total gene count in COGNATE); (4) average coding size/gene in exonic bp (mean added CDS length per transcript in COGNATE); (5) coding amount (total added length of all CDSs in COGNATE); (6) the average exon size in bp (mean exon length in COGNATE); (7) the average intron size in bp (mean intron length in COGNATE); (8) intron number (total intron count in COGNATE); (9) intron amount (total added length of all introns in COGNATE). Please note that we applied the same parameter terminology in the figure as Elliott and Gregory [4]. Values of these parameters were taken from the supplement of [4], including all genomes of the original set and partitioned by kingdoms (animals, red; land plants, orange; fungi, light blue; ‘protists’, dark blue). Values referring to all genomes are depicted by a black line. The plot shows the amount of missing data, i.e., for each parameter, the count of missing values per count of potential values was determined. Thus, 0% of missing data means that all values of the genome set under scrutiny were present, as is nearly the case for GC content. bp: basepairs; CDS: CoDing Sequence; Mb: Megabases

Another problem of terminological and methodological nature is the widespread use of means as descriptive summary statistic. Since many gene structure features are not normally distributed within a genome, the mean is an inappropriate summary statistic of these features. Yet, in many investigations, only the mean is calculated as a summary statistic of gene structure features (see [4] as well as the publications cited therein). Doing so can bias analyses and severely mislead comparisons between genomes, especially when one is represented by a mean, the other by a median. To illustrate this, we used results of COGNATE from analyzing the latest gene set of *Apis mellifera* (see above) and compared the obtained values of mean and median of exon size and intron size per transcript, respectively (Fig. 3, data in Additional file 3). In normally distributed data, means and medians are expected to be (nearly) identical, which is clearly not the case in *A. mellifera*. COGNATE calculates both means and medians for a wealth of parameters.

A third example of unclear usage of terms relates to the evaluation of intron density. The two above evaluated parameters — exon size and intron size per transcript — together with intron density per transcript can be understood as a proxy for gene structure, as demonstrated by Yandell et al. [1], and are thus of great interest in structural gene characterization. Note however that intron density as calculated by Yandell et al. [1] relates to protein length (i.e., count of introns/protein length). We advocate (and implemented in COGNATE) the relation of intron density to gene length as described above, since proteins as well as mature mRNAs are spliced and thus intron-free.

Aside from reporting important insights, Elliott & Gregory [4] advocated the need for standardization in large-scale comparisons of genomes. The inevitable problems of analyzing datasets with missing data could, in the future, be extenuated by a common, comprehensive set of basic parameters published together with genomic data. When publishing a genome and its annotation of protein-coding genes, it would be most beneficial to attach the complete set of COGNATE results to it to avoid problems resulting from changing versions of genomes and/or annotations. A set of standard metrics to advance standardization of parameter publishing was proposed by Elliott & Gregory [4], including “details of base pair composition, gene number, intron number and size, total repeat content, and TE abundance, diversity and activity” ([4], p. 8). Many other parameters can and should be used to describe the features of a genome completely, most of which go beyond the scope of COGNATE (e.g., properties of repetitive elements). Regarding protein-coding genes, we suggest to cover the descriptive parameters more broadly and to provide the following parameters as a minimum:

- assembly size (i.e., total added length of all SCSs, with and without Ns),
- assembly GC content (with and without ambiguity),
- gene count,
- median transcript length (tallying one representative transcript per gene),
- median CDS length,
- median CDS count per transcript (i.e., density),
- median CDS length per gene (i.e., coverage),
- coding amount (i.e., total added length of all CDSs),
- intron count,
- median intron length,
- median intron count per transcript (i.e., density),
- median intron length per gene (i.e., coverage),
- intron amount (i.e., total added length of all introns).

Following the establishment of standard parameters of gene model properties and the institution of a standard tool to acquire these, the next desirable step is the constitution of a “curated, user-friendly, open-access database [to] make this information accessible and usable in large-scale comparative analyses” ([4], p. 8).

Finally, we would like to draw the readers’ awareness also to a frequently encountered problem in comparative genomics: the source of primary sequence data or the version of gene annotations are often not clearly stated, which hampers reproducibility of the published analyses. Therefore, we emphasize the need for disclosing used databases, genome versions, and other source information in combination with data and results.

## Conclusion

Comparative meta-analyses of gene and genome characteristics, testing, for example, whether potential proteome diversity is reliably reflected by the total amount of coding sequences [31], rely on descriptive statistics of primary genome sequences and gene annotations. However, comprehensive standard statistics of genome organization and gene structure have not been fully or consistently defined with the effect that they are inconsistently collected or often incomplete. Due to this problem, comparative meta-analyses of gene and genome characteristics can be severely handicapped and are potentially unreliable. Obviously, this problem can be solved with the routine application of standard tools. The here presented software COGNATE allows effortless and flexible parameter disclosure as well as genome comparisons within its designated scope. Its merits include the comprehensive evaluation of an extensive set of standard and non-standard parameters of protein-coding genes, the provision of both primary data and summary statistics, and the use of explicit term definitions. COGNATE was developed in the hope to further promote and ease comparative studies, which should eventually yield insights into the evolution of genomes and gene repertoires.

## Availability and requirements

COGNATE is provided as a package, including source code, helper scripts (e.g., to check the presence of required Perl libraries), example data, GAL libraries, and manual at the ZFMK website and together with this publication as Additional file 4.

- **Project name**: COGNATE
- **Project home page**: https://www.zfmk.de/en/COGNATE and https://github.com/ZFMK/COGNATE
- **Operating system(s)**: platform independent
- **Programming language**: Perl
- **Other requirements**: GAL libraries (included)
- **License**: GNU GPLv3

The datasets analyzed during the current study are available in the NCBI RefSeq repositories^7,8^ and from the supplement^12^ of [4].

## Additional files

Additional file 1: Parameter table. List of parameters recorded by COGNATE. The first sheet of this table contains all 296 parameters evaluated by COGNATE, including the output file in which to find them and explanatory comments. Sorting for parameters, the individual feature (‘of’) or the feature location (‘per’), and files allows to quickly find a parameter of interest. The second sheet contains a comparison of the values recorded by COGNATE when analyzing the latest annotation of the *Apis mellifera* genome (genome version 4.5^8^, annotation release 103^9^) to those values given in the publications of the official gene sets version 1 [24] and 3.2 [25] and in the annotation report by NCBI^11^. As an addition, we included the results of GenomeTools’ ‘gt stat’ command applied to the annotation release 103 GFF file for comparison. (XLSX 43 kb)

Additional file 2: Definition table. Glossary and definitions used by COGNATE. This document contains the definitions used by COGNATE and in this manuscript for structural entities and measured parameters. Where available, we added matching Sequence Ontology terms. (PDF 110 kb)

Additional file 3: Result table. COGNATE results of analyzing exon and intron lengths of *Apis mellifera.* This data sheet contains the mean and median lengths of exons and introns, which are part of the 10,733 transcripts analyzed by COGNATE (default run, i.e., using the longest of each gene’s alternative transcripts). In total, 76,276 exons and 65,543 introns were taken into account. The data is visualized in Fig. 2. (XLSX 225 kb)

Additional file 4: The COGNATE package. This archive file contains the COGNATE package, including Perl scripts, Additional file 1: Parameter table, Readme, example data and output, and the GAL library. (ZIP 566 kb)

## Abbreviations

Bp: Nucleotide basepairs
CDS: Coding sequence
CpG o/e: Cytosine-guanine dinucleotides observed/expected
GC: Guanine, cytosine
Mb: Megabases (1 Mb = 1000 basepairs [bp])
MiB: Megabinary byte (1 MiB = 1,048,576 bytes)
RAM: Random access memory (‘working memory’ of a computer)
SCS: Scaffold or contig sequence
UTR: Untranslated region

## Footnotes

1 Standage DS. AEGeAn: an integrated toolkit for analysis and evaluation of annotated genomes. 2010–2015. http://standage.github.io/AEGeAn. Last accessed 20 March 2017.

2 GitHub: Holmes I. gfftools. 2011. https://github.com/ihh/gfftools. Last accessed 20 March 2017.

3 The BioPerl Project. 2016. http://bioperl.org. Last accessed 20 March 2017.

4 The Generic Model Organism Database: GFF format definition. 2016. http://gmod.org/wiki/GFF3. Last accessed 20 March 2017.

5 The Genome Annotation Library. 2016. http://www.sequenceontology.org/software/GAL.html. Last accessed 20 March 2017.

6 Data of *Ixodes scapularis*: NCBI: FTP directory of the Ixodes scapularis genome version JCVI_ISG_i3_1.0 and the corresponding protein-coding gene annotation (NCBI RefSeq). 2017. ftp://ftp.ncbi.nlm.nih.gov/genomes/all/GCF/000/208/615/GCF_000208615.1_JCVI_ISG_i3_1.0/. Last accessed 20 March 2017.

7 L90pcG, the count of SCSs necessary to cover 90% of the annotated protein-coding genes in an assembly, is here, to our knowledge, explicitly termed for the first time. Similar metrics have been described in other publications, for example, the “number of whole genome CARs [Contiguous Ancestral Regions] that cover 90% of one-to-one orthologous families” ([32], SOM, page 57). Although the notation of L for a number (instead of a length) appears to be counter-intuitive, we deliberately decided to follow the already established convention of N50 and L50, with N50 designating “maximum length L such that 50% of all nucleotides lie in contigs (or scaffolds) of size at least L” [33], and L50 designating the “number of sequences evaluated at the point when the sum length exceeds 50% of the assembly size” (Bradnam K. ACGT. 2015. http://www.acgt.me/blog/2015/6/11/l50-vs-n50-thats-another-fine-mess-that-bioinformatics-got-us-into. Last accessed 23 May 2017).

12 Elliott TA, Gregory TR. Supplement 1 – Genome data used in the analyses. 2015. http://rstb.royalsocietypublishing.org/highwire/filestream/32237/field_highwire_adjunct_files/0 /rstb20140331supp1.xlsx. Last accessed 20 March 2017.

8 NCBI: FTP directory of the *Apis mellifera* genome version 4.5 (NCBI RefSeq). 2016. ftp://ftp.ncbi.nlm.nih.gov/genomes/all/GCF /000/002/195/GCF_000002195.4_Amel_4.5/. Genome file downloaded 31 August 2015. Last accessed 20 March 2017.

9 NCBI: FTP directory of the *Apis mellifera* annotation release 103. 2017. ftp://ftp.ncbi.nlm.nih.gov/genomes/Apis_mellifera/GFF/. Annotation file downloaded 20 March 2017. Last accessed 20 March 2017.

10 NCBI: NCBI *Apis mellifera* Annotation Release 103 report site. 2016. https://www.ncbi.nlm.nih.gov/genome/annotation_euk/Apis_mellifera/103/. Last accessed 20 March 2017.

11 The Sequence Ontology. 2016. http://www.sequenceontology.org. Last accessed 20 March 2017.
