# Supplementary material for: COGNATE: comparative gene annotation characterizer
Source: BMC Genomics. 2017 Jul 17;18:535. doi: 10.1186/s12864-017-3870-8 (PMC5513398; doi:10.1186/s12864-017-3870-8)
Supplement: Supplementary file 2 — Definition table. Glossary and definitions used by COGNATE. This document contains the definitions used by COGNATE and in this manuscript for structural entities and measured parameters. Where available, we added matching Sequence Ontology terms. (PDF 110 kb) [file 12864_2017_3870_MOESM2_ESM.pdf]

Term<br>(Structural entities)	COGNATE definition	Sequence ontology term [1]
Genome assembly	Reconstructed DNA sequence(s) of the genome of an organism. Assembled by aligning and/or by scaffolding DNA sequence reads that are typically shorter than the natural intact DNA molecule(s).	**SO:0000353 sequence_assembly**:<br>A sequence of nucleotides that has been algorithmically derived from an alignment of two or more different sequences.<br>**SO:0001248 assembly:**<br>A region of the genome of known length that is composed by ordering and aligning two or more different regions.<br>**SO:0001876 partial_genomic_sequence_assembly:**<br>A partial DNA sequence assembly of a chromosome or full genome, which contains gaps that are filled with N's.
Structural gene annotation	Hypothesis on the locations of genes and gene features within a genome assembly. In case of protein-coding genes, the following features are typically annotated: start codon, stop codon, transcripts / mRNAs, exons, introns, untranslated regions (UTRs), coding sequences (CDSs).<br>Often, computer-inferred *de novo* annotations give the same start coordinate for a given gene, its mRNA, its start codon, its first CDS, and its first exon. The same applies to the end coordinate, being the same for a gene, its mRNA, its last CDS, its last exon, and its stop codon. However, this coincidence can be an artifact of gene prediction and does not necessarily always occur.<br>Structural predictions are derived from similarity of sequence, sequence motifs, structures, and/or manual curation.<br><br>Other (non-coding) genes may be structurally annotated in a similar manner with their applicable features.	
Functional gene annotation	Hypothesis on the functions of genes. Functional predictions are derived from experimental evidence and/or similarity of the nucleotide (or encoded amino acid) sequence, domains, sequence motifs, and/or structure of the genes or their respective proteins.	

**Term (Structural entities)**	**COGNATE definition**	**Sequence ontology term [1]**
SCS (Scaffold or Contig Sequence)	A contiguous sequence derived from a sequence assembly process. Can be a contig (derived from overlapping/alignment of reads) or a scaffold (derived from distance information, i.e., mapping, paired-end, or mate-pair data). A scaffold typically contains gaps (blocks of 'N's).	**SO:0000149 contig:**<br>A contiguous sequence derived from sequence assembly. Has no gaps, but may contain N's from unavailable bases.<br>**SO:0000148 supercontig[1]:**<br>One or more contigs that have been ordered and oriented using end-read information. Contains gaps that are filled with N's.<br>**SO:0000719 ultracontig:**<br>An ordered and oriented set of scaffolds based on somewhat weaker sets of inferential evidence such as one set of mate pair reads together with supporting evidence from ESTs or location of markers from SNP or microsatellite maps, or cytogenetic localization of contained markers.
Protein-coding gene	Region of DNA including all sequence elements necessary to encode a protein. The gene region of protein-coding genes in eukaryotes usually encompasses regulatory sequences up- and downstream, exons (including UTRs and CDSs [2]), and introns. The protein is produced by transcribing the exons and introns (results in a transcript), by splicing (removing introns) and/or otherwise modifying this transcript (results in mature mRNA), and translating the CDS of the resulting mRNA into an amino acid sequence.	**SO:0000001 region**:<br>A sequence_feature with an extent greater than zero. A nucleotide region is composed of bases and a polypeptide region is composed of amino acids.<br>**SO:0005836 regulatory_region**:<br>A region of sequence that is involved in the control of a biological process.<br>**SO:0000704 gene**:<br>A region (or regions) that includes all of the sequence elements necessary to encode a functional transcript. A gene may include regulatory regions, transcribed regions and/or other functional sequence regions.<br>**SO:0001217 protein_coding_gene**: - [2]

1: SO:0000148 matches the common definition of scaffold. The Sequence Ontology does not offer a term 'scaffold'.

2: No definition given for SO:0001217.

Term<br>**(Structural entities)**	**COGNATE definition**	**Sequence ontology term [1]**
Transcript	An RNA synthesized on a DNA or RNA template by an RNA polymerase [SO:0000673]. In case of protein-coding genes, the RNA includes all exons (i.e., the 5' and the 3' UTRs and all CDSs) and all introns of the gene; the transcript requires modifications (RNA maturation) to be ready for translation into a protein. The transcript thus represents pre-mRNA.	**SO:0000673 transcript**:<br>An RNA synthesized on a DNA or RNA template by an RNA polymerase.<br>**SO:0000185 primary_transcript**:<br>A transcript that in its initial state requires modification to be functional.
mRNA<br>(messenger RNA)	Transcript of a protein-coding gene that has been post-transcriptionally modified. In eukaryotes, the modification typically includes 5'-capping, polyadenylation, and the splicing (removal) of introns, which can result in alternative transcripts (recombination of all or some exons). Naturally occurring mature mRNA typically consists of a 5' cap, 5' UTR, concatenated CDSs, 3' UTR, and a poly-A tail. In structural annotations, 5' cap and poly-A-tail are not indicated.<br>Only the concatenated CDS between start codon and stop codon is translated into amino acid sequence.	**SO:0000233 mature_transcript:**<br>A transcript which has undergone the necessary modifications, if any, for its function. In eukaryotes this includes, for example, processing of introns, cleavage, base modification, and modifications to the 5' and/or the 3' ends, other than addition of bases. In bacteria functional mRNAs are usually not modified.<br>**SO:0000234 mRNA**:<br>Messenger RNA is the intermediate molecule between DNA and protein. It includes UTR and coding sequences. It does not contain introns.
Exon	Any part of a gene that becomes part of the mature mRNA. Exons can be classified by their position within the mRNA and contain UTRs and CDSs in various combinations [2]. Thus, neither all exons nor all parts of them are necessarily coding. In structural annotations, UTRs and exons may be separately annotated and not overlapping; in these cases, exons coincide with CDSs.	**SO:0000147 exon**:<br>A region of the transcript sequence within a gene which is not removed from the primary RNA transcript by RNA splicing.
CDS<br>(CoDing Sequence)	Any part of a gene that becomes translated, i.e., contains information for synthesizing an amino acid sequence [4]. All CDSs are exonic [2].	**SO:0000195 coding_exon**:<br>An exon whereby at least one base is part of a codon (here, 'codon' is inclusive of the stop_codon).<br>**SO:0000316 CDS**:<br>A contiguous sequence which begins with, and includes, a start codon and ends with, and includes, a stop codon.
Intron	Every transcribed non-coding (in respect of this gene) part of a gene that is removed by RNA splicing during RNA maturation.	**SO:0000188 intron**:<br>A region of a primary transcript that is transcribed, but removed from within the transcript by splicing together the sequences (exons) on either side of it.

Term<br>(Structural entities)	COGNATE definition	Sequence ontology term [1]
UTR<br>(UnTranslated Region)	mRNA sequence of a protein-coding gene that is non-coding (i.e., remains untranslated) and lies 5'- or 3'-adjacent to sequences in the same mRNA that are translated (CDSs). UTRs are parts of exons [2].	**SO:0000203 UTR**:<br>Messenger RNA sequences that are untranslated and lie five prime or three prime to sequences which are translated.

Term (Measured parameters)	Definition
GC content	Amount of guanine and cytosine in a given DNA sequence, in percent.
GC content without ambiguity	Amount of guanine, cytosine, and S (G or C IUPAC ambiguity base) in a given DNA sequence, excluding ambiguous bases (NRYKMBDHV), in percent.
CpG o/e	CpG dinucleotide depletion, normalized by the GC content of the region under scrutiny. The CpGo/e for each sequence is defined as the frequency (count/total length) of CpG dinucleotides divided by the product of the frequencies of C nucleotides and G nucleotides in the sequence [3]
Length	Total count of nucleotide bases/amino acids in a DNA/protein sequence, respectively.
Count	Total count of features.
Coverage	Ratio of the length of a feature covered by another, length-wise. For example, 'exon coverage of a transcript' translates to the added length of all exons divided by the length of their corresponding annotated transcript.
Density	Ratio of the count of features found along another feature. E.g., the count of exons divided by the length of their corresponding annotated transcript.

References

[1] The Sequence Ontology Browser. http://www.sequenceontology.org/browser/obob.cgi. Accessed 15 November 2016.

[2] Zhang MQ. Computational prediction of eukaryotic protein-coding genes. Nature Reviews Genetics. 2002;3:698–709.

[3] Elango N, Hunt BG, Goodisman MAD, Yi SV. DNA methylation is widespread and associated with differential gene expression in castes of the honeybee, Apis mellifera. PNAS. 2009;106:11206–11.

[4] Mudge JM, Harrow J. The state of play in higher eukaryote gene annotation. Nature Reviews Genetics. 2016;17:758–72.
